# The Functions of the Semi-Circular Canals

**Published:** 1878-08

**Authors:** H. Gradle

**Affiliations:** Lecturer on Physiology


### Original Lectures.

### THE FUNCTION OF THE SEMI-CIRCULAR CANALS.

A LECTURE DELIVERED AT THE CHICAGO MEDICAL COLLEGE.

BY H. GRADLE,

LECTURER ON PHYSIOLOGY.

While studying the perception of sounds, we found in the cochlea an apparatus answering all requirements, which the theory of acoustics demands for the accomplishment of that purpose. There remains for consideration the other portion of the labyrinth—the utricle and semi-circular canals.

What rôle these structures play in the perception of sound, we are unable to say; neither reasoning from physical premises nor experimental data justify the assertion that they are at all necessary for hearing. In some animals, the cochlea as well as the semi-circular canals can be extirpated separately; while loss of the former results in total deafness, destruction of the latter does not impair audition perceptibly. Still, both the constant occurrence of the semi-circular canals throughout the vertebrate series and their peculiarly complicated structure, lead us to attribute to them some important duty.

With the exception of some of the lowest fishes, where only one (Myxinoids) or two (Petromyzon) rings are found, all vertebrate animals present the same general arrangement of the semi-circular canals. It is also remarkable to observe how little this apparatus varies in actual size amongst the different animals. Three hollow rings, or rather portions of three rings, are joined in such a manner as to communicate with a central hollow receptacle—the utricle. In the higher animals, in which the inside of the petrous bone consists of spongy tissue, this tissue becomes compact in forming the walls of the labyrinth.

The three rings are arranged in such a manner, that their planes coincide with the three dimensions of space. We have thus one horizontal and two vertical canals. Of the latter, one corresponds to the sagittal plane of the head, and the other to the frontal plane. It seems, therefore, preferable to name the canals according to their position, instead of using the somewhat confusing anatomical nomenclature. The three canals communicate with the cavity of the utricle through five openings; the posterior end of the sagittal and the upper end of the frontal canal unite into one opening. Each canal presents a dilatation at one of its ends, the ampulla; this is situated in front on the horizontal and sagittal canals, below on the frontal canal. Within the bony walls we find an accurate cast, so to speak, of their form in the membranous labyrinth. The latter, however does not fill out the entire cavity enclosed by the bony walls, and is hence in contact with the bony wall at one spot only, leaving a free space between the remainder of its circumference and the lining periosteum. This space containing the so-called perilymph deserves to be considered as a lymphatic space, since Schwalbe proved its continuity with the subdural spaces. The membranous labyrinth may be described as consisting of three coats; an outer fibrous coat continuous with the periosteum, a middle hyaline tunic with villi-like projections into the interior, and lastly an internal lining of pavement epithelium. The epithelium, however, is altered in the ampullæ. In each ampulla we find a ridge, the crista auditiva, and a similar crest exists also in the utricle—the macula auditiva, which is lined with cylindrical epithelium instead of pavement cells. Between the cylinder cells there exist fusiform cells, evidently also of epithelial nature, each surmounted with a single hair, which hair passes as a central axis through the entire cell, and can be shown to be in continuity with a nerve fibre. This is probably the most positive instance of a nerve termination in epithelial cells, of which we are aware. The nerves in question are derived from the vestibular branch of the auditory nerve, which sends one twig to each ampulla, one to the macula, and one to the sacculus. The interior of the membranous labyrinth contains the endolymph. The otoliths floating in this fluid are limited in their motions by a gelatinous matrix.

Mere anatomical considerations do not inform us of the possible uses of this apparatus ; fortunately, however, it is open to experimentation. Some fifty years ago the first experimental results were described by Flourens in a manner not since excelled. The animal mainly employed by Flourens and subsequent observers, was the pigeon in which the semi-circular canals can be easily exposed and experimented upon.

After exposing the bone and removing the external plate, the canals are seen in the midst of the spongy tissue. Care must be taken to avoid hemorrhage from the vessels accompanying the canals ; besides, there is danger of wounding the utricle and the aquæductus vestibuli. The latter, very large in this bird, was described by Schklarewsky as the "cavitas mesootica," containing a process of the cerebellum ; but this has not been confirmed.

Let us follow the details of a well performed section of the horizontal canal. A mere wound of the bony wall is not followed by the characteristic symptoms which are due to section of the membranous canal. The latter is quite sensitive, its section giving rise to pain. After the operation the bird is seen to swing its head frequently in a horizontal direction, but this is not very noticeable unless the animal is excited. Gradually this tendency ceases. After the lapse of some weeks it is no longer observed.

Another noticeable result is the impairment of walk and flight. The animal does not move in a straight line, especially when frightened will it show movements of *manege*, turning in a circle *towards* the side of the operation. No paresis can be found, but the foot of the operated side is not as steady in its movement as the other. As a matter of difficult explanation, the impairment is sometimes more noticeable in flight, sometimes in walking. It diminishes, however, in the course of time.

When both horizontal canals are divided the result is considerably intensified. The swinging of the head, scarcely noticeable in the former case is now a prominent symptom. At the least excitation, often even without such, the animal will shake its head violently in the horizontal plane, usually slowly and with wide excursion. But as the excitement increases, the movement often changes to a mere rapid oscillation. The shaking of the head occurs even after extirpation of the cerebral hemispheres; it is prevented, however, by deep narcosis. In the course of a few weeks these movements lose in intensity but they do not disappear as long as the bird survives, which, in some cases has been for many months.

A bilateral operation intensifies also the impairment of walking. On attempting to walk, the bird turns to one, or to the other side in such a manner as to prevent it from reaching its object and even escaping from its pursuer. Although the bird can use its wings, continuous flight has become impossible. When thrown up in the air it describes zigzag curves until it strikes against some object and falls to the ground. Time, however, will materially improve locomotion.

On dividing the vertical canals vomiting often occurs, otherwise the results are similar though not the same as after section of the horizontal canals. If the operation be done on one frontal canal, swinging of the head is at once observed. But the swinging is not in a horizontal direction, but obliquely, corresponding to the plane of the divided canal. Otherwise it resembles the swinging after section of one horizontal canal. The impairment of walk is also slightly different. There is a tendency to turn towards the operated side; besides, however, the animal is in danger of falling over—backwards if the frontal canal be divided—forwards when the operation occurred on the sagittal canal.

An operation on both sides is more indicative of the canals divided, than when it is limited to one side alone. The tendency to somersaults renders the animal more helpless than the movements of *manege* alone. The swinging of the head is always in the plane of the divided canals, with an apparent exception. Section of both sagittal canals, will result in a vertical up and down movement, as well as section of both frontal canals. In the pigeon, however, the vertical canals do not coincide exactly with the planes of the head, but are set at an angle of 20 to 30 degrees. Hence the two sagittal canals are not parallel, but divergent, and the same is true of the two frontal canals. Mechanically, therefore, the left frontal and the right sagittal canals are really mates (and conversely), and this view is justified by the experiment. On dividing the frontal canal of one side, and the sagittal of the other, the swinging occurs in the oblique direction corresponding to their plane. Finally, on dividing the horizontal and vertical canals, the head describes a figure 8 in swinging, and the highest degree of incoordination is obtained.

We have thus described the constant results of a well-performed section of one or more canals, and you see them illustrated in the birds now before you. But these results are very easily complicated by the consequences of wounding of the utricle or aquæductus vestibuli. While in the former case, the animal, when undisturbed, will show no signs of the operation except the shaking of the head, the mutilation of the utricle or aquæduct betrays itself by an inability to maintain the balance under all conditions. The bird finds it necessary to use its head or beak for support in order to avoid falling. Every movement threatens to overthrow the dizzy animal; it performs a variety of the most incoordinate attempts to retain its equilibrium. As a rule, the pigeon must be fed; it cannot coordinate the movements of its head sufficiently to pick up its food. Nevertheless, the animal has lost none of its skill in cleaning itself; with its beak and its foot it can reach every part of its body with accuracy. The swinging of the head is very violent during this state, but no regular direction is maintained, as after a simple section. Usually the head is kept in a peculiar position, the occiput turned until it reaches almost to the ground, while the beak is turned upward and backward. This is not due to muscular paralysis; on the contrary, it is caused by a contracture, and hence an intense narcosis allows the head to return to its normal position. That this malposition of the head increases the unsteadiness of the animal, was shown by Goltz by suturing the head of normal pigeons to the breast, with the result of producing a similar want of balance. But Goltz claims too much when he tries to account for the incoordination by this contracture of the muscles of the neck. Section of the canals alone seldom gives rise to this contracture, although it does so occasionally after some time, and then quite suddenly. In such cases an extension of an inflammatory process to the cerebellum is usually found.

In mammals, section of the semi-circular canals is quite difficult, on account of their protected position. It has, however, been performed with success by Flourens in very young rabbits. The impairment in walking, the movements of *manege* and the tendency to turn over were met with as in pigeons. The swinging movements, however, pertained more to the muscles of the eye than the head. On the other hand, the experiment will also succeed in frogs. In this animal, the muscular incoordination is noticeable, but the swinging of the head or turning of the eyes is not observed.

Whatever animal be employed, the result of operations on the semi-circular canals is always an appearance of dizziness, which increases with the extent of the lesion. That the term "dizziness" is the proper one can be shown especially on pigeons with extended mutilations. The animal, tumbling with every step it attempts, and performing wild movements to retain its balance, is easily quieted by a gentle support, and all voluntary movements cease when the hand of the observer assists it in maintaining its balance. But the various phenomena will appear clearer to you after studying the appearances of dizziness in man.

A simple way to render yourself dizzy is to whirl around rapidly on one foot. The consequences of this rotation were described in detail over fifty years ago by Peorkinye. Recently, however, such experiments have been repeated with a greater show of accuracy by making use of disks rotated by machinery without jarring, on which the observer sits in a chair. I refer to the experiments performed by the physicist Mach, and independently of him by Mr. Crum Brown.

These observers found that with closed eyes, and on a smoothly running apparatus, rotation around a vertical axis is at once perceived. They could recognize besides the direction, rapidity and approximately the angular extent of the turning. A perfectly uniform rotation, however, soon ceases to be perceptible, and the observer believes himself at rest. But as soon now as the actual rotation ceases, a feeling of turning in the opposite direction is experienced. If the apparatus had been revolving from *left to right*, the observer, after the apparatus had come to a standstill, believes himself turning from *right to left;* and this impression continues some time, according to the rapidity of the original rotation. That the perception of the rotation, as well as the after-impression, if we choose to call thus the imaginary rotation, is obtained through some sensory apparatus situated in the head, is shown by two observations. On the one hand, the delicacy with which we perceive the extent and rapidity of the passive rotation varies with the position of the head. Secondly, however, the direction of the apparent rotation changes also with movements of the head. If, while rotating from left to right, the head is inclined horizontally on the right shoulder, the after-impression on coming to rest will be an apparent movement from right to left, *i. e.*, in the direction from the forehead towards the chin of the head inclined laterally. If now the head is raised vertically to its ordinary position, the observer seems to tumble over forwards. In other words, the plane and direction of the apparent rotation have become altered in relation to the body but have not changed in relation to the head.

We can, furthermore, produce a feeling of rotation by passing a galvanic current through the head, and this result is obtained most readily by applying the poles to the mastoid fossæ. These points of application are evidently the most favorable for the passage of the current through the labyrinth and its nerves.

When an apparent or (as Crum Brown calls it) complementary rotation is observed after cessation of actual motion, no disagreeable dizziness is, as a rule, felt, unless the eyes be opened. In the latter case all external objects seem to have a motion in the opposite direction of the original rotation, *i. e.*, in the direction of the complementary rotation. This peculiar *vibratory* motion of external objects is the main cause of the dizziness. A similar dizziness can in fact be produced during absolute rest by moving the eyeball rapidly to and fro with the fingers, when the real motion of the eye (which, however, is not the result of voluntary contraction of the ocular muscles) is falsely interpreted as an apparent motion of external objects.

Motion of external objects, or in other words, their displacement in relation to our body, can be perceived in a two-fold manner, either by the motion of the retinal image or by the ocular movements necessary to retain the retinal image in its original place. But to distinguish between real motion of external objects and passive motion of our own body is more than a mere visual impression, it is a matter of judgment. During passive rotation objects do not seem to change their place, at least at first. On observing a person revolving around his vertical axis peculiar ocular movements are noticeable. If he turns from left to right, his eyes are seen to turn at the same rate from right to left, so as to maintain their original direction. But the deviation to the left soon becomes excessive and with a sudden rapid jerk the eyes are turned towards the right. Again they wander towards the left as long as possible, when the former movement is repeated. In other words, the eyes are turned slowly against the direction of rotation, but rapidly in that direction. The image of the object gazed upon in the first place is thus retained in its original retinal position, until the eye performs the rapid movement in the direction of the rotation. The ocular movement requisite to maintain the original gaze is correctly interpreted as being necessitated by the bodily rotation. The displacement, however, which the retinal image suffers during the rapid turn in the direction of the bodily motion is ignored, as we habitually disregard all apparent motion of external bodies due to rapid ocular movements. The ocular movements therefore are *compensatory*, enabling us to recognize the real static condition of external objects.

If the passive rotation of the body continues, the ocular muscles become fatigued and the compensatory movements cease. The displacement of retinal images is now interpreted as real motion of external objects. The same result may be obtained at once by directing our eyes steadily towards the finger held immovably during the rotation of the body, when of course compensatory movements are also excluded, and objects seem to move in the opposite direction. The compensatory ocular movements, although recognized by our judgment are by no means voluntary; they occur during blindness as well as when the eyes are closed. In the latter case they may be felt through the closed lids.

When the actual rotation of the body has ceased and the apparent rotation in the opposite direction is observed, the play of the ocular movements is inverted. If the original motion of the body was towards the right and the apparent rotation towards the left, the eyes are turned during this apparent rotation *slowly* towards the right and whirled *rapidly* to the left. Hence external objects seem to move in the direction of the apparent rotation. Ocular movements of this nature occur also during the feeling of apparent rotation caused by the passage of the galvanic current.

Besides the ocular movements compensatory activity of the muscles of the trunk and limbs is also observed during apparent rotation. If the body is not supported when real rotation ceases, the danger of falling in the direction of the apparent rotation is imminent; the floor seems to cave in, and to guard against this, muscular efforts are made to restore the bodily equilibrium. Exactly this condition is witnessed also when the head is galvanized.

The compensatory movements of the limbs as well as the eyes are in neither case voluntary, but purely reflex in their nature.

In animals, also, compensatory movements occur during rotation, actual and apparent. In the rabbit the movements are mostly ocular, although the muscles of the neck are also called into requisition; but where the mobility of the eye is so limited, as in pigeons, the compensatory movements consist only in deviations of the head. While holding this pigeon with its head forward I shall turn around from left to right. You can notice how its head deviates towards its left side, until it is suddenly jerked to its extreme right, when it again wanders toward the left. What we accomplish by ocular movements the bird obtains through the free mobility of its neck. When the real rotation ceases, an apparent rotation is evidently felt by the animal, but with this slow motion its objective signs are scarcely perceptible. If, however, the animal is whirled around by machinery, its dizziness betrays itself by the very same phenomena as those *which are seen after section of the semi-circular canals.* The pigeon shakes its head laterally, obliquely or vertically, according to the axis, around which it was whirled, and turns around rapidly to one or the other side; the rabbit presents the characteristic oscillations of its eyes, while performing exquisite movements of *manege* or rolling over in somersaults according to the direction of the original motion. *In fact as long as the dizziness persists, the animals cannot be distinguished from the victims of section of*

*some of the semi-circular canals.* We have called these compensatory movements reflex and the proof can be furnished that one of their starting points is in the labyrinth. If the eyes of a normal pigeon are covered with a cap, the compensatory movements still continue during passive revolution. If, however, the canals and vestibule are extirpated on both sides, no further movements will occur during rotation, when the eyes are closed. (Breuer.) As long as the eyes are open, however, the movements are excited by sight. The importance of the labyrinth is further shown by the results of section of the auditory nerve, or since this is difficult, by extirpation of the vestibule and canals. The ability to maintain the bodily equilibrium is completely destroyed by this operation. On the other hand, excitation of one auditory nerve produces violent ocular movements and bodily rotation in the direction towards the opposite side. (Cyon.)

Pathological accidents present us occasionally with a similar condition in man. We owe to Menière the first description of a state of dizziness, increased by every movement, and attended with involuntary compensatory efforts, which is due to lesions of the semi-circular canals. Often, however, no lesion is found in these structures, but in other parts of the ear. But a mere strong contraction of the tensor tympani muscle will suffice to press the stapes against the oval window, and thus increase the intra-labyrinthine pressure, and necessarily irritate the nerve terminations. The dizziness sometimes caused by washing out the external meatus is probably due to a reflex contraction of this muscle.

Summing up our evidence, we can therefore state that:

1. We are able to perceive the direction, rapidity and angular extent of a passive rotation.

2. The organ for this perception is situated in the head.

3. Reflex compensatory movements of eyes (and other muscles) occur during a passive rotation, for the purpose of maintaining the equilibrium of the body and the correct appreciation of the static condition of external objects.

4. These compensatory movements do not occur when the vestibules and canals are extirpated and the eyes closed.

5. Lesions of the semi-circular canals are followed by phenomena identical with those seen during the dizziness after a rapid rotation.

In short, we are forced to recognize in the semi-circular canals an organ for the perception of rotatory motion.

We can explain the perception of rotation by a simple physical hypothesis proposed by Breuer. When a vessel containing a fluid is put into motion, the inertia of the fluid has to be overcome before the fluid molecules follow the motion of the vessel. Hence, *when there are no resisting walls*, a temporary current, backward in relation to the motion of the vessel, is established by the inertia of the fluid molecules. This current continues until the internal friction has absorbed the original momentum due to the inertia of the fluid. The semi-circular canals, although not complete rings anatomically, yet contain a circle of fluid, being completed by the utricle. In such a ring-shaped tube a lateral motion can produce no current on account of the resisting walls, but no obstacle exists to a current whenever a rotation occurs in the plane of the ring; in other words, whenever the ring rotates around its imaginary axis. Since the planes of the rings coincide with the three dimensions of space, no rotation of the head can occur, without producing a current of inertia in one or more rings. With a uniform rotation the current speedily exhausts itself, and the fluid moves uniformly with its walls. This agrees with our sensations; a uniform passive rotation soon ceases to be perceptible.

When the motion of the body ceases, a current is produced in the opposite direction, as compared with the direction of the first current. The inertia keeps up the movement of the endolymph in the direction of the original rotation; the wall of the canal, however, is at rest, and hence the relative direction of the current is inverted. This again explains fully the inverted direction of the apparent rotation; for the perception of these currents no more delicate apparatus can be conceived than the fine nervous hairs of the fusiform epithelial cells. The hairs, being bathed by the endolymph, must be deflected by every current, and the impulse of the current is probably heightened by the vibration of otoliths. The structure of the hairs will besides overthrow the validity of an objection on physical grounds. It may be claimed, that on account of the narrow caliber of the canals, the current of inertia cannot correspond in duration and intensity to the subjective sensation. But the retarded return to the straight position of these delicate hairs can easily account for the prolonged sensation.

Breuer raises the question whether each ampulla can perceive the current in either direction, and attempts to solve it in the positive. He claims, that after extirpation of the apparatus o one side, compensatory movements will still occur in either direction. Moreover he attempted to prove it directly. Opening carefully the bony canal, he pressed on the uninjured membranous canal with some dull object (a piece of paper), thus creating a current in the direction from the canal toward the ampulla. The result was a movement of the head in the plane of the canal and in the direction of the ampulla. On the other hand, when a current in the opposite direction was established by puncturing the membranous canal with a fine needle to permit the exit of the endolymph, a movement of the head took place also in the plane of the canal, but in the direction from the ampulla toward the canal. This difficult experiment however did not succeed often. The result of a *section* of a single canal is somewhat different. The section, in fact, produces not only a current through the exit of the endolymph, but also a considerable tearing of and traction on the membranous canals. In the beginning of the lecture I did not describe minutely the slight movements subsequent to a one-sided operation, but as Flourens stated, and Breuer confirmed, the section of a canal gives rise always to a shaking of the head in the plane of the injured canal, but from the central position toward the normal side. Within some minutes, however, these movements are reversed, and the head is now thrown toward the side of the operation. Breuer suggests that this reversal of the movements may be due to a state of tonus or constant slight stimulation of the normal labyrinth (perhaps from the constant secretion of endolymph). The irritation from the wounding of one canal having abated, the tonus from the other side might now predominate, and hence the reversal of the movements. This reversal in the direction of the shaking of the head does, indeed, not occur when the vestibule and canals of one side have been extirpated some time previous to the division of a canal of the other side.

BIBLIOGRAPHY.

*Flourens.*—Rechêrches exp. sur les Propriétés et les Fonctions du Syst. Neıveux, 1842, p. 464.

*Flourens.*—Comptes Rendus, 1861.

*Harless.*—Wagner's Handwörterbuch d. Phys. Bd. iv, p. 422.

*Czermak.*—Comptes Rendus, 1860.

*Czermak.*—Jenaische Zeitschrift iii, 1867.

*Vulpian.*—Phys. Compare du Syst. Nerveux, 1866, p. 600.

*Schiff.*—Physiolog. des Menschen, 1859, p. 399.

*Brown-Séquard.*—Phys. and Pathology of the nervous system, p. 194.

*Goltz.*—Pflügers Archiv. f. Phys., iii, p. 172, 1870.

*Schklarewsky.*—Göttinger Nachrichten, 1872, No. 15.

*Waldeyer.*—Centralblatt f. d. med. Wiss., 1872, p. 385.

*Loewenberg.*—Centralblatt, 1873, p. 278.

*Boettcher.*—Kritische Bemerk. u. neue Beitr. zur Literatur d. Gehörlabyrinths, Dorpat, 1872.

*Boettcher.*—Arch. f. Ohrenheilkunde, Bd. 9, p. 1.

*Berthold.*—Arch. f. Ohrenhlkde, Bd. 9, p. 77.

*Curschman.*—Deutsche Klinik, 1874, No. 3.

*E. Cyon.*—Pflügers Archiv. Bd. viii, p. 306.

*Cyon.*—Comptes Rendus, April, 1876.

*Hitzig.*—Archiv. für Anat., 1871, p. 716.

*Mach.*—Wiener Academ. Sitzungsberichte, iii, Abth. lxviii, 124 and lxix 44, (Abstr. in Journ. of Anat. and Phys., Apr., 1876.)

*A. Crum Brown.*—Journ. of Anat., 1874, p. 327.

*J. Breuer.*—Wiener Med. Jahrbücher, 1874, I. Heft, p. 72, and 1875. I. Heft, p. 87.

---

VOMITING IN PREGNANCY. — Some years ago the author reported some cases of chorea rapidly cured by ether spray. He now resorts to it in treating obstinate cases of this complaint. He uses Richardson's apparatus and directs the spray over the pit of the stomach, and the corresponding level of the spine, continuing it from three to five minutes or longer, and as often as every three hours. In rebellious cases he alternates with chloroform. The treatment is indicated as soon as there is any nausea. —*Gazette des Hôpitaux*, Apr. 20, 1878).

